# A70 EXPLORING THE ROLE OF MICROBIAL PHOSPHOLIPIDS IN VISCERAL HYPERSENSITIVITY AND ALTERED EMOTIONAL BEHAVIOUR INDUCED BY INFLAMMATORY BOWEL DISEASE (IBD)-ASSOCIATED MICROBIOTA

**DOI:** 10.1093/jcag/gwae059.070

**Published:** 2025-02-10

**Authors:** F A Vicentini, M Hall-Bruce, J Pujo, G H Rueda, A Nardelli, A Jiang, G De Palma, E Verdu, S Collins, P Bercik

**Affiliations:** McMaster University, Hamilton, ON, Canada; McMaster University, Hamilton, ON, Canada; McMaster University, Hamilton, ON, Canada; McMaster University, Hamilton, ON, Canada; McMaster University, Hamilton, ON, Canada; McMaster University, Hamilton, ON, Canada; McMaster University, Hamilton, ON, Canada; McMaster University, Hamilton, ON, Canada; McMaster University, Hamilton, ON, Canada; McMaster University, Hamilton, ON, Canada

## Abstract

**Background:**

IBD is characterized by relapsing episodes of inflammation in the gastrointestinal tract. Abdominal pain is common in IBD and often persists without overt inflammation. Moreover, patients with IBD often develop anxiety and depression, further impairing their quality of life. Recently, gut microbiota emerged as a tentative pathophysiological factor in pain and mood disorders. However, little is known about the specific mechanisms by which microbiota modulate pain and mental health in IBD.

**Aims:**

We hypothesized that bacteria contribute to visceral hypersensitivity and behavioural abnormalities in IBD via modulation of bioactive phospholipids, focusing on lysophosphatidylcholine (LPC) and its metabolite lysophosphatidic acid (LPA), which were previously linked to chronic pain, anxiety and depression.

**Methods:**

Fecal samples from healthy controls (HC n=15) and IBD patients (n=35; Crohn’s Disease (CD) n=18, Ulcerative Colitis (UC) n=17) were collected to assess the levels of LPC and LPA via mass-spectrometry. Samples from selected donors (HC n=5, CD n=6, UC n=5) were used to colonize germ-free C57BL/6 mice (n=115) of both sexes. After 3 weeks, the light preference and tail suspension tests were performed to assess anxiety- and depression-like behaviour. Visceral sensitivity was assessed by visceromotor responses to colorectal distension (100-300 μL). Gut inflammation was evaluated by histology. Microbiota composition was assessed via 16S rRNA gene sequencing.

**Results:**

Fecal concentrations of LPC and LPA were higher in patients with IBD, mainly in those with chronic abdominal pain. Mice colonized with IBD microbiota displayed a reduced preference for the light compartment and increased immobility, suggestive of anxiety- and depression-like behaviour, respectively. Mice with IBD microbiota had higher visceromotor responses to colorectal distension. Interestingly, we observed increased responses to non-noxious stimuli (100 μL), suggestive of visceral allodynia. Microbiota profiles differed between mice colonized with IBD or HC microbiota. Also, mice with increased visceral sensitivity had lower abundance of Bacteroidales taxa, which contains genes related to the catabolism of LPC and LPA. Histology analysis did not show signs of overt inflammation in the jejunum, ileum, or colon.

**Conclusions:**

Microbiota from patients with IBD negatively affect the gut-brain axis by inducing changes in anxiety- and depression-like behaviour and triggering visceral hypersensitivity. Bacterial production of LPC and LPA may be responsible for these deleterious effects.

**Funding Agencies:**

CCC, CIHRCrohn’s & Colitis Foundation

